# The Safety of Baduanjin Exercise: A Systematic Review

**DOI:** 10.1155/2021/8867098

**Published:** 2021-01-21

**Authors:** Jianqi Fang, Liying Zhang, Fangzhen Wu, Jiajia Ye, Shuhe Cai, Xiaowen Lian

**Affiliations:** ^1^Department of Rehabilitation, Fujian University of Traditional Chinese Medicine, Fuzhou, Fujian 350000, China; ^2^Department of Prevention and Health Care, Fujian University of Traditional Chinese Medicine Subsidiary Rehabilitation Hospital, Fuzhou, Fujian 350000, China; ^3^Department of Rheumatism and Immunity, The Second Affiliated Hospital of Fujian Traditional Chinese Medical University, Fuzhou, Fujian 350000, China; ^4^Department of Rehabilitation Assessment, Fujian University of Traditional Chinese Medicine Subsidiary Rehabilitation Hospital, Fuzhou, Fujian 350000, China; ^5^Department of Orthopaedic Rehabilitation, Fujian University of Traditional Chinese Medicine Subsidiary Rehabilitation Hospital, Fuzhou, Fujian 350000, China; ^6^Fujian Key Laboratory of Rehabilitation Technology, 13 Hudong Road, Fuzhou, Fujian 350000, China

## Abstract

**Objectives:**

Baduanjin exercise is a form of Qigong exercise therapy that has become increasingly popular worldwide. The aims of the current systematic review were to summarize reported adverse events potentially associated with Baduanjin exercise based on currently available literature and to evaluate the quality of the methods used to monitor adverse events in the trials assessed.

**Methods:**

The English databases PubMed, Cochrane library, and EMbase were searched from inception to October 2020 using the keywords “Baduanjin” or “eight session brocade.” Only studies that included Baduanjin exercise therapy were included.

**Results:**

Forty-seven trials with a total of 3877 participants were included in this systematic review. Twenty-two studies reported protocols for monitoring adverse events, and two studies reported the occurrence of adverse events during training. The adverse events reported included palpitation, giddiness, knee pain, backache, fatigue, nervousness, dizziness, shoulder pain, chest tightness, shortness of breath, and muscle ache.

**Conclusions:**

Only two studies reported adverse events that were potentially caused by Baduanjin exercise. Adverse events related to Baduanjin exercise in patients with chronic fatigue syndrome may include muscle ache, palpitation, giddiness, knee pain, backache, fatigue, nervousness, dizziness, shoulder pain, chest tightness, and shortness of breath. Further studies conducted in accordance with the Consolidated Standards of Reporting Trials statement guideline incorporating monitoring of adverse events are recommended. Additional clinical trials in which Baduanjin exercise is used as a main intervention are needed, and further meta-analysis may be required to assess its safety and reach more informed conclusions in this regard in the future.

## 1. Introduction

Qigong exercise is a core part of traditional Chinese medicine therapy that has existed for more than 2000 years [1]. Baduanjin is one of the traditional Chinese Qigong exercise therapies of mild to moderate intensity, and it is considered to be an effective approach to promoting health [2]. It emphasizes the mind-body connection, slow movements while breathing deeply, and muscle stretching with mental concentration; it also has profound therapeutic effects in patients with various medical conditions [3–8]. Although Baduanjin has been practiced for thousands of years in China, globally it is not as popular as Tai Chi [1, 9]. To date, no review has systematically investigated the safety of Baduanjin.

The reporting of adverse events (AEs) is vital when introducing and evaluating a new therapy. An AE has been defined as any unwanted experience during a study regardless of whether the AE was directly related to an intervention [10]. Standard reporting of AEs is suggested in the Consolidated Standards of Reporting Trials (CONSORT) statement [11]. It is strongly recommended that AEs should be described in the Results sections of published reports [12]. Despite existing guidelines on the reporting of AEs [11], the degrees to which AEs are described in experimental studies are still inadequate [13, 14]. Insufficient reporting of AEs may lead to skepticism, criticism, and even rejection of a therapy.

To address the above-described research gaps with respect to Baduanjin exercise, a systematic review of the available evidence on its safety is needed. Accordingly, the main purpose of the present systematic review was to investigate and summarize the AEs reported in previous studies that included Baduanjin exercise. A secondary aim was to evaluate the quality of the methods used to monitor AEs in the studies assessed.

## 2. Methods

### 2.1. Guideline Adherence and Eligibility Criteria

The current analysis was conducted in accordance with the Preferred Reporting Items for Systematic Reviews and Meta-Analyses (PRISMA) guidelines. Only studies published in English were included. Studies involving patients with medical conditions and studies using healthy participants were included. No restrictions on sociodemographic characteristics were applied. Experimental studies that included any type of Baduanjin exercise were included. No restrictions on the types of Baduanjin interventions or control interventions were applied.

### 2.2. Types of Outcome Measures

The definition of an AE was any undesirable experience during the study period. AEs were categorized as serious, non-serious, and intervention-related using a prior meta-analytic review as a guide [15]. Serious AEs were defined as those associated with hospitalization or medical or surgical needs, and those that were potentially life-threatening or resulted in death. Other AEs were defined as non-serious.

### 2.3. Search Methods

The PubMed, Cochrane library, and Embase electronic databases from inception to October 2020 were searched. The search string used to search PubMed and Embase was Baduanjin [Title/Abstract] OR eight session brocade [Title/Abstract]. The search string used to search the Cochrane library was Baduanjin [Title/Abstract/Keyword] OR eight session brocade [Title/Abstract/Keyword].

Initial screening to exclude duplicate and irrelevant studies based on article titles was conducted by the first author (Fang). The abstracts of the remaining studies were then independently reviewed by two authors (Fang and Zhang). The remaining full texts were then reviewed by two different independent authors with reference to the eligibility criteria (Wu and Ye). Only research articles that met the selection criteria were included in the subsequent review and assessment. The References sections of relevant articles were also reviewed by the authors. Consensus was reached via discussion if there were disagreements between two reviewers. Only reports published in English were included in the analysis [16].

### 2.4. Data Extraction and Management

Data extraction from the reports identified included characteristics of the timing, frequency, and types of AEs reported based on the Extension of the CONSORT statement [11], and it was performed by both Fang and Zhang independently. All authors participated in discussion to reach a consensus in cases of disagreements. In two cases in which researchers had not clearly described AEs in their published articles, we emailed the corresponding authors seeking further details on intervention-related AE data and baseline data. Only one of these corresponding authors emailed us back in response to our questions. All of the authors of the current study discussed the AEs that remained unclear in the second study due to the lack of a response from the corresponding author, and made collective judgments with respect to those AEs.

## 3. Results

### 3.1. Reports Identified

**Figure 1:**
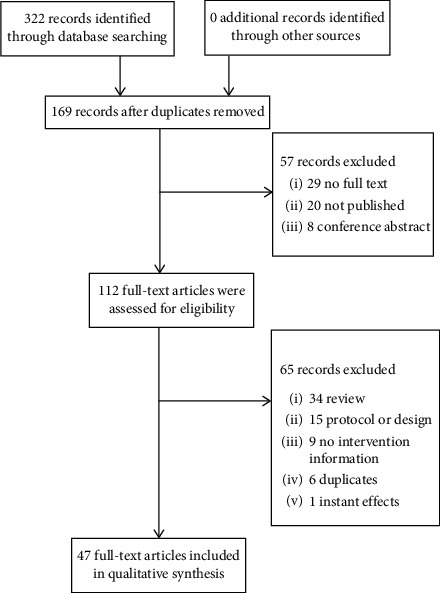
The flow of the literature search.

A total of 322 records were initially identified. Of these, 112 full-text articles were subsequently obtained based on the predetermined selection criteria, and a total of 47 articles were ultimately included in the current review. The flow of the literature search is presented in Figure 1.

### 3.2. Characteristics of Participants and Settings

A combined total of 3877 participants were included in the trials assessed in the final analysis, 2343 women and 1234 men; 3 studies did not report the exact number of males and females [17–19]. The mean ages in individual studies ranged from 20.8 to 83.0 years, and the median mean age was 60.1 years (interquartile range 49.9–65.5 years). The percentages of female participants in the studies ranged from 0% to 100%, and the median was 66.1% (interquartile range 38.7–80.2%).

The sample sizes in the reports ranged from 1 to 271, and the median was 68 (interquartile range 42–110). Most of the studies were conducted in China, including ten in Fujian [20–29], six in Beijing [19, 30–34], five in Taiwan [35–39], five in Guangdong [7, 40–43], four in Hong Kong [6, 44–46], three in Shanghai [18, 47, 48], two in each of Hebei [49, 50], Jiangsu [51, 52], Sichuan [53, 54], and Hubei [17, 55], and one in each of Tianjin [4], Shanxi [56], Jiangxi [57], and Macau [58]. One study was conducted in Singapore [59], and one study was conducted in the USA [60].

Eleven trials included healthy participants only [17, 20, 22, 30, 33, 36, 37, 39, 49, 55, 60], and the remaining 36 included participants with a variety of physical and mental health conditions [4, 6, 7, 18, 19, 21, 23–29, 31, 32, 34, 35, 38, 40–48, 50–54, 56–59].

### 3.3. Characteristics of Intervention Groups and Control Groups

The Baduanjin exercise regimes used in the studies varied in content, frequency, duration, and intensity. The majority of studies used standing Baduanjin exercises as an intervention [4, 6, 17–39, 41–59], two studies used a mixed model including standing and sitting Baduanjin exercises [40, 60], and one study used sitting Baduanjin exercises only [7]. The duration of Baduanjin exercise ranged from 12 minutes to 120 minutes. The frequency of sessions varied from once per week to 14 times per week, with overall exercise programs lasting from 8 weeks to 1 year. Five studies did not report the qualifications of instructors [17, 34, 40, 57, 58].

Of the 47 eligible studies, 8 evidently did not include controls [7, 28, 38, 39, 48, 58–60], and 5 used more than two control groups [21–24, 49]. Three studies used walking as a control [34, 42, 54], and other control measures used in single studies were muscle relaxation training [30], reading [6], physical therapy [19], and an aerobic exercise [43]. Twenty-seven studies used non-interventional controls, including usual care [4, 17, 18, 20, 25–27, 29, 31–33, 35–37, 40, 41, 44–47, 50–53, 55–57]. Further details of the characteristics of the studies are presented in Table 1.

### 3.4. Patterns of AEs

Of the 47 studies assessed, 22 reported protocols used to monitor AEs [4, 6, 17, 20, 21, 26, 27, 29, 32, 35, 37, 38, 40, 41, 43, 44, 46–48, 52, 55, 59], but only 2 reported the actual occurrence of AEs during the trial [44, 52]. Of these two studies, one reported muscle ache only [52] and the other reported muscle ache, palpitation, giddiness, knee pain, backache, fatigue, nervousness, dizziness, shoulder pain, chest tightness, and shortness of breath [44] (Table 2).

## 4. Discussion

Baduanjin exercise is becoming increasingly popular around the world as it has been associated with therapeutic benefits for various medical conditions. It is now offered in hospital and community settings across China to reduce clinical symptoms and improve quality of life. The risk of harm from Baduanjin exercise may be minor, as suggested by the current systematic review, and older adults or patients with chronic illness are more likely to experience benefits associated with its clinical effects and affordability.

In the present systematic review, the reporting of AEs in Baduanjin exercise studies was insufficient, and this limits the conclusions that can be drawn to date about its safety [10]. A total of 47 studies with a combined total of 3877 participants were included in the current systematic review. Of the 47 studies, 22 reported the utilization of protocols to monitor AEs, and 2 reported the actual occurrence of AEs. The AEs reported included palpitation, giddiness, knee pain, backache, fatigue, nervousness, dizziness, shoulder pain, chest tightness, shortness of breath, and muscle ache. Some studies reported AEs at the end of the study, which may have resulted in recall bias. More frequent measures to identify AEs, and the incorporation of multiple modalities via which to report AEs throughout the study (interviews, questionnaires, and tests), may contribute to the generation of more reliable evidence [12].

None of the studies in the current systematic review reported any serious AEs. The most commonly reported AE related to Baduanjin exercise was muscle ache [44, 52], which is consistent with previous observations in other exercise studies [61–65]. Notably, some Baduanjin exercises entail a semi-squat position, for example, “Session 2, Open the Arms as an Archer Shooting Both Left- and Right-Handed”; “Session 5, Sway the Head and Shake the Tail”; and “Session 7, Grip the Palms to Improve Strength” [25]. In such sessions, participants are required to coordinately move upper limbs and trunks while in a semi-squat position, which may greatly improve muscle strength, particularly that of the lower extremities. These types of Baduanjin exercises may have contributed to the mild muscle aches reported during the first 2 weeks of practice [44, 52].

In one trial that included participants with chronic fatigue syndrome (CFS), 24 AEs were reported after Baduanjin exercise [44], which is consistent with other physical exercise studies in individuals with CFS [66]. This suggests that patients with CFS may experience some non-serious AEs after standard Baduanjin exercises. Notably however, that report [44] only mentions the monitoring of AEs in the intervention group, and there is no information regarding AEs in the control group. Further research is needed that incorporates the monitoring and reporting of all AEs in both intervention and control groups based on the CONSORT statement, to facilitate informative conclusions in patients with CFS.

The current study had several limitations. Only reports published in English were included. Additional potentially relevant studies may have been conducted that were published in other languages. Secondly, the number of trials included in the review was small, and they only employed descriptive statistics and summaries of AEs. Further meta-analyses assessing AEs potentially associated with Baduanjin exercise may be conducted.

## 5. Conclusion

Estimation of any potential harm related to a novel therapy is a vital consideration when promoting that therapy. Poor reporting of AEs may substantially limit the conclusions that can be drawn relating to Baduanjin exercise. The number of trials with strict reporting of AEs is small. One of the studies in the current analysis suggests that AEs related to Baduanjin exercise in patients with CFS may include muscle ache, palpitation, giddiness, knee pain, backache, fatigue, nervousness, dizziness, shoulder pain, chest tightness, and shortness of breath. It is recommended that in future studies AEs are rigorously monitored and strictly reported in accordance with the CONSORT guideline. Further meta-analysis may enhance understanding of the safety of Baduanjin exercise in the future.

## Figures and Tables

**Table 1:** Characteristics of included studies.

Source	Area	Average age (age range)	Study population	No. of men/women	Intervention group	Control group
An et al. [47]	Shanghai	65 ± 7.36	Patients with KOA	0/28	*N* = 14 Baduanjin 30 min; 5 times/w; 8 w	*N* = 14 keep routine as usual 8 w

An et al. [48]	Shanghai	65.2 ± 7.3	Patients with KOA	3/19	*N* = 22 Baduanjin 30 min; 5 times/w; 1y	—

Bao et al. [60]	American	83	Older adults	3/13	*N* = 16 Baduanjin (mixed) 45–50 min; 12 w	—

Chan et al. [44]	HK	39 ± 7.93	Women with chronic fatigue syndrome-like illness	42/108	*N* = 75 Baduanjin 90 min; 16 times/9 w (group training) + ≥30 min; 7 times/w; 9 w (self- practice)	*N* = 75 keep routine as usual 9 w

Chan et al. [45]	HK	32.5–47.0	Persons with chronic fatigue syndrome-like illness	0/108	*N* = 46 Baduanjin 90 min; 16 times/9 w (group training) + ≥30 min; 7 times/w; 9 w (self-practice)	*N* = 62 keep routine as usual 9 w

Chen et al. [36]	Taiwan	45.16 ± 5.78	Healthy middle-aged women	0/87	*N* = 44 Baduanjin 3 times/w; 12 w	*N* = 43 keep routine as usual 12 w

Chen et al. [37]	Taiwan	71.75 ± 8.13	Community-dwelling older people	19/36	*N* = 27 Baduanjin 30 min; 3 times/w; 12 w	*N* = 28 keep routine as usual 12 w

Chen et al. [53]	Sichuan	60.10 ± 7.06	Patients with chronic obstructive pulmonary disease	147/85	*N* = 117 usual care + Baduanjin 30 min; 7 times/w; 3 m	*N* = 115 usual care 3 m

Chen et al. [7]	Guangdong	62	Patients with dysfunctional ventilatory weaning response	1/0	*N* = 1 usual care + Baduanjin (sitting Baduanjin) 30 min; 2 times/d; 19 d	—

Chen et al. [38]	Taiwan	38.9 ± 9.6	People with severe mental illness	8/3	*N* = 11 Baduanjin 90 min; 2 times/w; 8 w	—

Chen et al. [30]	Beijing	22.5 ± 2.0	Healthy college students	16/26	*N* = 21 Baduanjin 90 min; 5 times/w; 8 w	*N* = 21 muscle relaxation training 90 min; 5times/w; 8 w

Chen et al. [35]	Taiwan	70.29 ± 13.53	Patients with stable heart failure	42/38	*N* = 39 Baduanjin 35 min; 3 times/w; 12 w	*N* = 41 keep routine as usual 12 w

Chen et al. [40]	Guangdong	60.7 ± 11.12	Patients with AMI after PCI	59/23	*N* = 43 usual care + Baduanjin (mixed) 30 min; 2 times/d; 3 d (sitting Baduanjin) 30 min; 5 times/w; 4 d-24 w (standing Baduanjin)	*N* = 39 keep routine as usual 24 w

Cheung et al. [46]	HK	41.75 ± 8.99	Women survivors of intimate partner violence	0/271	*N* = 136 Baduanjin 120 min; 2 times/w; 6 w (group training) + 60 min; 1 time/w; 7–22 w (group follow-up) +30 min; 7 times/w; 1–22 w (self-practice)	*N* = 135 keep routine as usual 22 w

Dong et al. [57]	Jiangxi	30–65	Patients with phlegm-dampness hypertension	26/21	*N* = 23 Baduanjin 60 min; 5 times/w; 16 w	*N* = 24 usual care 16 w

Fan et al. [41]	Shenzhen	71.1 ± 6.3	Elderly patients with sleep disturbances	34/105	*N* = 67 Baduanjin 45 min; 5 times/w; 24 w	*N* = 72 keep routine as usual 24 w

Han et al. [31]	Beijing	56 ± 8.86	Postoperative non-small cell lung cancer patients	28/32	*N* = 30 Baduanjin 60 min; 3 times/w; 1 w (training); 30 min; 10 times/w; 2 w-3 m (self-practise)	*N* = 30 keep routine as usual 3 m

Hsu et al. [39]	Taiwan	49.93 ± 4.38	Middle-aged women	0/31	*N* = 31 Baduanjin 20 min; 3 times/w; 12 w	—

Jin et al. [17]	Wuhan	34.2 ± 14.57	Physically healthy people	Not mentioned	*N* = 55 Baduanjin 30–60 min; ≥3 times/w; 16 w	*N* = 55 keep routine as usual 16 w

Jing et al. [49]	Tangshan	75.08 ± 5.26	Elderly housebound	40/78	*N* = 39 Baduanjin 60–90 min; 2 times/m; 3 m 1times/m; 3–6 m	*N* = 79 control group1: CBT 6 m control group2: Baduanjin + CBT 60–90 min; 2 times/m; 3 m 1 time/m; 3–6 m

Li et al. [55]	Wuhan	34.2 ± 14.6	Physically healthy adults	36/74	*N* = 55 Baduanjin 30–60 min; 3 times/w; 16 w	*N* = 55 keep routine as usual 16 w

Li et al. [20]	Fuzhou	20.78 ± 1.1	Healthy college students	36/170	*N* = 101 Baduanjin 60 min; 5 times/w; 12w	*N* = 105 keep routine as usual 12w

Li et al. [54]	Sichuan	50.98 ± 7.76	Patients with schizophrenia	47/14	*N* = 30 Baduanjin 40 min; 5 times/w; 24 w	*N* = 31 brisk walking 40 min; 5 times/w; 24 w

Liang et al. [42]	Guangdong	55.25 ± 9.38	Patients with essential hypertensive	38/22	*N* = 30 usual care + Baduanjin 20 min; 10 times/w; 6 m	*N* = 30 usual care + walking 20 min; 10 times/w; 6 m

Liao et al. [32]	Beijing	18–60	People with fatigue- Predominant subhealth	33/96	*N* = 62 Baduanjin 30 min; 14 times/w; 6 w	*N* = 67 keep routine as usual 6 w

Liu et al. [56]	Shanxi	57.2 ± 5.4	Women with diabetes	0/35	*N* = 17 Baduanjin 90 min; 6 times/w; 24 w	*N* = 18 keep routine as usual 24 w

Liu et al. [21]	Fuzhou	59.17 ± 7.36	Patients with KOA	25/83	*N* = 29 Baduanjin 60 min; 5 times/w; 12 w	*N* = 79 control group1: Taichi 60 min; 5 times/w; 12 w control group2: cycling 60 min; 5 times/w; 12 w control group3: health education 60 min; 1 time/w; 12 w

Liu et al. [59]	Singapore	77.1 ± 5.9	Frail older adults	3/9	*N* = 12 Baduanjin 90 min; 2 times/w; 4 w (training) 90 min; 3 times/w; 5–16 w (self-practise)	—

Lu et al. [51]	Nanjing	55.11 ± 11.51	Patients with colorectal cancer undergoing chemotherapy	56/31	*N* = 43 Baduanjin 20–40 min; ≥5 times/w; 24 w	*N* = 44 usual care 24 w

Mao et al. [43]	Beijing	60.86 ± 10.63	Patients after acute myocardial infarction	73/37	*N* = 56 Baduanjin 30 min; 2 times/w; 12 w	*N* = 54 aerobic exercise 30 min; 2 times/w; 12 w

Tao et al. [22]	Fuzhou	61.29 ± 4.85	Older adults	20/41	*N* = 15 Baduanjin 60 min; 5 times/w; 12 w	*N* = 46 control group1: Taichi 60 min; 5 times/w; 12w control group2: keep routine as usual 12 w

Tao et al. [23]	Fuzhou	65.55 ± 4.40	Patients with mild cognitive impairment	18/39	*N* = 20 Baduanjin 60 min; 3 times/w; 24 w	*N* = 37 control group1: brisk walking 60 min; 3 times/w; 24w control group2: health education 30 min/w; 24 w

Tsang et al. [6]	HK	80.11 ± 5.63	Chinese depressed elders with chronic illness	12/26	*N* = 21 Baduanjin 45 min; 3 times/w; 12w	*N* = 17 reading 45 min; 3 times/w; 12 w

Wang [33]	Beijing	59.35 ± 1.6	Old people	42/71	*N* = 55 Baduanjin 60 min; 5–7 times/w; 6 m	*N* = 58 keep routine as usual 6 m

Wang et al. [18]	Shanghai	65.19 ± 4.88	Patients with essential hypertensive	Not mentioned	*N* = 61 usual care + Baduanjin 20–30 min; 4-5 times/w; 1 y	*N* = 61 usual care 1 y

Wang et al. [4]	Tianjin	54.09 ± 7.76	Breast cancer survivors	0/86	*N* = 46 Baduanjin ≥20 min; 7 times/w; 6 m	*N* = 40 Keep routine as usual 6 m

Xia et al. [24]	Fuzhou	65.51 ± 4.35	Older adults with mild cognitive impairment	23/46	*N* = 23 Baduanjin + health education Baduanjin:60 min; 3 times/w; 24w + Health education:30 min; 1 time/8 w; 24 w	*N* = 46 control group1: brisk walking + health education brisk walking:60 min; 3 times/w; 24w + health education:30 min; 1 time/8 w; 24 w control group2: keep routine as usual + health education 30 min; 1 time/8 w; 24 w

Xiao et al. [19]	Beijing	67.8 ± 9.4	Patients with Parkinson's disease	Not mentioned	*N* = 35 Baduanjin 60 min; 4 times/w; 6 m	*N* = 33 physical therapy 6 m

Xiao et al. [34]	Beijing	67.53 ± 8.56	Patients with idiopathic Parkinson's disease	67/29	*N* = 48 Baduanjin + walk Baduanjin:12–15 min; 4 times/w; 6m + walk: ≥30 min; 7 times/w; 6 m	*N* = 48 walk ≥30 min; 7 times/w; 6 m

Xiao et al. [50]	Hebei	Not mentioned	Adults with cardiovascular diseases	50/79	*N* = 66 Baduanjin 24 min; 5 times/w; 16 w	*N* = 63 Keep routine as usual 16 w

Xie et al. [52]	Nanjing	37.39 ± 11.35	Patients with ankylosing spondylitis	35/11	*N* = 23 Baduanjin 2times/w; 4 w (instruction) ≥3 times/w; 5–12 w (self-practise)	*N* = 23 Keep routine as usual 12 w

Ye et al. [25]	Fuzhou	63.78 ± 6.39	Patients with KOA	25/25	*N* = 25 Baduanjin 40 min; 3 times/w; 12 w	*N* = 25 Keep routine as usual 12 w

Ye et al. [26]	Fuzhou	64.36 ± 5.34	Older adults with KOA	19/37	*N* = 28 Baduanjin 40 min; 3 times/w; 12 w	*N* = 28 Keep routine as usual 12 w

Zhang et al. [58]	Macau	24 ± 7	Women with premenstrual syndrome	0/40	*N* = 40 Baduanjin (8 sessions) 14 times/w; 3consecutive menstrual cycles	—

Zheng et al. [28]	Fuzhou	60	Elderly Population at risk for Ischemic stroke	11/9	*N* = 20 Baduanjin 30 min; 3-5 times/w; 12 w	

Zheng et al. [27]	Fuzhou	60.14 ± 6.3	Community adults at risk of ischamic stroke	61/109	*N* = 85 Baduanjin 60 min; 5 times/w; 12 w	*N* = 85 keep routine as usual 12 w

Zheng et al. [29]	Fuzhou	62.19 ± 7.87	Patients with post-stroke cognitive impairment	41/7	*N* = 24 usual care + Baduanjin 40 min; 3 times/w; 24 w	*N* = 24 usual care 24 w

HK: Hong Kong; KOA: knee osteoarthritis; CBT: cognitive behavioral therapy; min: minutes; w: week; m: month; y: year; N/No: number; AMI: acute myocardial infarction; PCI: percutaneous coronary intervention.

**Table 2:** The types of adverse events in both groups.

Source	Tre	Tse	Tnon	Cre	Cse	Cnon	Tdropout	Cdropout	F-U
An et al. [47]	0	0	0	0	0	0	3	4	4 m
An et al. [48]	0	0	0	—	—	—	6	—	No
Bao et al. [60]	Unclear	Unclear	Unclear	—	—	—	9	—	No
Chan et al. [44]	24 muscle ache	0	4 palpitation; 3 giddiness; 2 knee pain; 2 backache; 2 fatigue; 2 nervousness; 2 dizziness; 1 shoulder pain; 1 chest tightness; 1 shortness of breath	0	0	0	18	17	No
Chan et al. [45]	Unclear	Unclear	Unclear	Unclear	Unclear	Unclear	0	0	3 m
Chen et al. [17]	Unclear	Unclear	Unclear	Unclear	Unclear	Unclear	1	2	No
Chen et al. [37]	0	0	0	0	0	0	1	0	No
Chen et al. [53]	Unclear	Unclear	Unclear	Unclear	Unclear	Unclear	3	5	No
Chen et al. [7]	—	—	—	—	—	—	—	—	No
Chen et al. [38]	0	0	0	—	—	—	5	—	8 w
Chen et al. [30]	Unclear	Unclear	Unclear	Unclear	Unclear	Unclear	0	0	No
Chen et al. [35]	0	0	0	0	0	0	9	8	No
Chen et al. [40]	0	0	0	0	0	0	5	9	No
Cheung et al. [46]	0	0	0	0	0	0	16	8	No
Dong et al. [57]	Unclear	Unclear	Unclear	Unclear	Unclear	Unclear	2	3	No
Fan et al. [41]	0	0	0	0	0	0	5	9	No
Han et al. [31]	Unclear	Unclear	Unclear	Unclear	Unclear	Unclear	No	No	No
Hsu et al. [39]	Unclear	Unclear	Unclear	—	—	—	0	—	No
Jin et al. [17]	0	0	0	0	0	0	0	0	No
Jing et al. [49]	Unclear	Unclear	Unclear	Unclear	Unclear	Unclear	1	1	No
Li et al. [55]	0	0	0	0	0	0	9	0	No
Li et al. [20]	0	0	0	0	0	0	8	4	12 w
Li et al. [54]	Unclear	Unclear	Unclear	Unclear	Unclear	Unclear	0	0	0
Liang et al. [42]	Unclear	Unclear	Unclear	Unclear	Unclear	Unclear	No	No	No
Liao et al. [32]	0	0	0	0	0	0	2	0	12
Liu et al. [56]	Unclear	Unclear	Unclear	Unclear	Unclear	Unclear	3	2	No
Liu et al. [21]	0	0	0	0	0	0	6	26	No
Liu et al. [59]	0	0	0	—	—	—	1	—	No
Lu et al. [51]	Unclear	Unclear	Unclear	Unclear	Unclear	Unclear	2	1	No
Mao et al. [43]	0	0	0	0	0	0	2	3	No
Tao et al. [22]	Unclear	Unclear	Unclear	Unclear	Unclear	Unclear	10	4	No
Tao et al. [23]	Unclear	Unclear	Unclear	Unclear	Unclear	Unclear	3	9	No
Tsang et al. [6]	0	0	0	0	0	0	2	2	No
Wang [33]	Unclear	Unclear	Unclear	Unclear	Unclear	Unclear	No	No	No
Wang et al. [18]	Unclear	Unclear	Unclear	Unclear	Unclear	Unclear	No	No	No
Wang et al. [4]	0	0	0	0	0	0	0	0	No
Xia et al. [24]	Unclear	Unclear	Unclear	Unclear	Unclear	Unclear	3	6	No
Xiao et al. [19]	Unclear	Unclear	Unclear	Unclear	Unclear	Unclear	14	16	No
Xiao et al. [34]	Unclear	Unclear	Unclear	Unclear	Unclear	Unclear	3	4	No
Xiao et al. [50]	Unclear	Unclear	Unclear	Unclear	Unclear	Unclear	1	4	No
Xie et al. [52]	7 muscle ache	0	0	0	0	0	7	7	No
Ye et al. [25]	Unclear	Unclear	Unclear	Unclear	Unclear	Unclear	0	0	No
Ye et al. [26]	0	0	0	0	0	0	0	0	No
Zhang et al. [58]	Unclear	Unclear	Unclear	—	—	—	9	—	No
Zheng et al. [28]	Unclear	Unclear	Unclear	—	—	—	0	—	No
Zheng et al. [27]	0	0	0	0	0	0	6	16	No
Zheng et al. [29]	0	0	0	0	0	0	5	8	4 w

Abbreviations. Tre: related adverse events of intervention group; Tse: serious adverse events of intervention group; Tnon: non-serious adverse events of intervention group; Cre: related adverse events of control group; Cse: serious adverse events of control group; Cnon: non-serious adverse events of control group; Tdropout: dropouts of intervention group; Cdropout: dropouts of control group; F-U: follow-up; m: month; w: week.

## Data Availability

The datasets used and analyzed during the current study are available from the corresponding author upon reasonable request.

## Supplementary Materials

Supplementary Materials The supplementary data file includes the comparison list of PRISMA Checklist.
